# Characterization and zoonotic impact of Shiga toxin producing *Escherichia coli* in some wild bird species

**DOI:** 10.14202/vetworld.2017.1118-1128

**Published:** 2017-09-24

**Authors:** Hanaa Mohamed Fadel, Rabab Afifi, Dheyazan Mohammed Al-Qabili

**Affiliations:** 1 Department of Animal Hygiene and Zoonoses, Faculty of Veterinary Medicine, Suez Canal University, Ismailia, Egypt; 2 Department of Wildlife and Zoo Medicine, Faculty of Veterinary Medicine, Suez Canal University, Ismailia, Egypt; 3 Department of Veterinary Public Health, Agriculture and Veterinary Medicine College, Thamar University, Yemen

**Keywords:** antibiotic, *eae*, random-amplified polymorphic DNA polymerase chain reaction, Shiga toxin producing *Escherichia coli*, *stx*_1_, *stx*_2_, wild birds

## Abstract

**Aim:**

Wild birds are considered silent vectors of some zoonotic water and food borne pathogens of public health significance. Owing to the importance of Shiga toxin producing *Escherichia coli* (STEC) as the most pathogenic among the emerging diarrheagenic *E. coli* groups that can infect man; the present study was designed to detect the occurrence of STEC among wild birds in Egypt.

**Materials and Methods:**

A total of 177 intestinal content swab samples originating from five wild bird species were investigated for the presence of *E. coli* and STEC by standard culture methods. Suspect STEC isolates were further characterized by serotyping, random amplified polymorphic DNA polymerase chain reaction (RAPD PCR), antimicrobial resistance pattern and PCR detection of *stx*_1_, *stx*_2_, and *eae* genes.

**Results:**

A total of 30 suspect STEC isolates from 30 positive birds’ samples were detected and identified on STEC CHROMagar (semi-captive pigeons, 15; house crows, 8; cattle egrets, 3; moorhens, 2; and house teals, 2). 25 isolates were grouped into 13 serogroups (O:20, O:25, O:26, O:27, O:63, O:78, O:111, O:114, O:125, O:128, O:142, O:153, and O:158), while five were rough strains. The distribution of STEC virulence genes among wild birds was as follows: 16 birds carried *stx*_1_ gene only (nine pigeons [28.1%], six crows [7.1%], and one cattle egret [5.6%]). *Stx*_1_ and *stx*_2_ genes together were detected in four birds (one cattle egret [5.6%], two moorhens [6.1%], and one house teal, [10%]). Only one pigeon (3.1%) possessed the three alleles. Disk diffusion test results showed that cefixime was the most effective against STEC serotypes with (93.3%) sensitivity, followed by gentamycin (56.7%), and amoxicillin (50%). On the other hand, all the recovered STEC isolates were resistant to cefotaxime, doxycycline, cephalothin, and sulfisoxazole. RAPD fingerprinting using primers OPA-2 and OPA-9 showed that STEC isolates were heterogeneous; they yielded 30 and 27 different clusters, respectively.

**Conclusions:**

Wild birds carry STEC and may add to the contamination of the surrounding environment.

## Introduction

Diarrheagenic *Escherichia coli* (DEC) strains are leading causes of diarrheal illnesses throughout the world [1,2]. There are five pathotypes (groups) of DEC. Shiga toxin-producing *E. coli* (STEC) is the only zoonotic among these groups. STEC are ubiquitous food and water borne pathogens inhabiting different animals, wildlife, humans as well as the environment [3-5]. STEC may also be referred to as verocytotoxin-producing *E. coli* (VTEC). They produce toxins that are variously known as verotoxins, verocytotoxins, or Shiga toxins. They are named for their similarity to the Shiga toxin produced by the bacterium *Shigella dysenteriae*. Enterohemorrhagic *E. coli* is a subset of VTEC that carries an additional virulence factor called intimin, which is encoded by the *eae* gene. Intimin assists in colonization and attachment to intestinal epithelial cells and effacement of microvilli. STEC strains are characterized by the production of one or both of two toxins, Shiga toxin 1 or Shiga toxin 2, which is encoded by the genes *stx_1_* and *stx_2_*. Combinations of these virulence genes (or their variants) are associated with life-threatening damage to major organ systems [5-7]. There are 300-400 known STEC serotypes; of which approximately 200 are able to cause disease in humans [8]. When infecting humans, they often cause bloody diarrhea, hemorrhagic colitis (HC), hemolytic uremic syndrome (HUS), and thrombotic thrombocytopenia purpura [4,5,8]. The most renowned example is *E. coli* O157:H7 which has been incriminated in human outbreaks since the 1980’s [9-12]. On the other hand, the recovery rate of non-O157:H7 STEC is the same or even exceeds that of O157:H7 [6]. The epidemiology of some STEC serotypes is well understood, while for others needs more investigation. The transmission routes of these pathogens may involve the direct fecal-oral route either from infected persons, animals, birds or by the consumption of contaminated food and water. The findings of Santaniello *et al*. [9], Cernicchiaro *et al*. [11], Nielsen *et al*. [13], Foster *et al*. [14], Persad and LeJeune [15], and Kobayashi *et al*. [16,17] lent support to the claim that wild birds are vectors and reservoirs for the maintenance and spread of STEC infections. The interactions between humans and wild birds are obvious; they reside in human habitats, migrate between waste collection areas, cattle, pig and poultry farms and deposit their droppings in soil and water and hence allowing the transmission of these zoonoses to man and animals [9,11,13-20]. The long survival time of STEC in soil (for up to 7 months) may give the opportunity for such transmission [10,21-23]. Furthermore, the emergence and dissemination of multi-drug resistant (MDR) bacteria in the environments constitute a global risk to human and animal health [24,25]. Aquatic wild birds, in particular, are often considered indicators for this environmental pollution [18,24,26]. Wild birds can serve as reservoirs of antibiotic resistant bacteria including *E. coli* and may contribute to the global spread of MDR *E. coli* in natural ecosystems [18,24,27,28].

After all, typing methods for discriminating different bacterial strains of the same species have become urgent epidemiological tools in disease prevention and control [29]. Traditional typing systems that are based on phenotypes, such as serotype, biotype, or antibiogram have been used for many years. However, other methods that examine the relatedness of isolates at a molecular level have reformed researchers’ capability to differentiate among bacterial types and subtypes [29]. One of the useful means that is used for this purpose is Random-Amplified Polymorphic DNA PCR (RAPD PCR) analysis. Unlike traditional polymerase chain reaction (PCR) analysis, RAPD analysis does not require any specific knowledge of the DNA sequence of the target organism. RAPD is an inexpensive and relatively powerful typing tool for many bacterial species. It has a high discriminatory capacity for typing *E. coli* isolates in the case of suspected cross infection or epidemic spread [30,31].

Due to the lack of information about the role of wild birds in the dissemination of STEC in the study area, this study was planned to elucidate the role of wild birds as reservoirs of antibiotic-resistant, *stx*- and *eae*-producing strains of STEC. The prevalence of these STEC strains in wild birds was examined using standard culture methods. STEC isolates were characterized by serotyping, antibiotic susceptibility, multiplex PCR, and RAPD PCR tests.

## Materials and Methods

### Ethical approval

The study protocol was approved by the Council of the Department of Animal Hygiene and Zoonoses.

### Sampling

The work was conducted in Egypt, namely, at Ismailia (Latitude: 30°36′15″ N and Longitude: 32°16′20″ E) and Damietta Cities (Latitude: 31°24′59″ N and Longitude: 31°48′47″E). 177 intestinal content swab samples originating from five wild bird species were collected over the years (2013 and 2016). They comprised (84) house crows (*Corvus splendens*), (33) moorhens (*Gallinula chloropus*), (32) semi-captive pigeons (*Columba livia*), (18) cattle egrets (*Bubulcus ibis*), and (10) house teals (*Anas crecca*). The moorhens were purchased from different retailers at live wild bird markets in Damietta City. Pigeons (semi-captive) and house teals were purchased from Ismailia’s live bird markets. Cattle egrets were hunted from different parks at Ismailia City using traps. A professional hunter was hired to shoot crows that were present near human residence areas in Ismailia City. The selected spp. were chosen because they either approach human habitats (pigeons, cattle egrets, and crows) or because they are commonly raised and/or consumed in the study area (pigeons, house teals, and moorhens). The birds’ handling, transportation and euthanization were performed in compliance with the American Veterinary Medical Association guidelines on the euthanasia of animals [32].

### Isolation and identification of E. coli and STEC

Dissection of the euthanized birds was undertaken under aseptic conditions. The intestine was opened, sterile cotton swabs were saturated with about 1 g of the intestinal contents and immediately put into sterile tubes containing 9 ml of 1% tryptone broth (Oxoid, Basingstoke, UK) and incubated at 37°C for 24 h. Two loopfuls of the incubated broth were aseptically streaked onto eosin methylene blue (EMB; Oxoid) and STEC CHROMagar™ (Paris, France) that were prepared according to manufacturers’ instructions and incubated at 40°C and 37°C, respectively, for 24-48 h. Two of suspect colonies on EMB that were green to deep red purple colored with a green metallic tinge sheen and those on STEC CHROMagar (entirely mauve, or mauve with white edge) were selected and biochemically identified according to USFDA [33].

### Serological identification of STEC isolates

*E. coli* isolates selected from STEC CHROMagar were serogrouped on the basis of their “O” antigen from the Reference Lab for Veterinary Quality Control on Poultry Production, Animal Health Research Institute, Dokki, Egypt. The identified isolate was preserved in tryptone broth 1% with adding glycerol to a final concentration of 15%. The tubes were kept at −20°C for further analysis.

### Antibiogram susceptibility pattern of STEC

Disk diffusion method was used to identify suspect STEC isolates. Seven antibiotics (Oxoid) that are commonly used in veterinary and human medicine were chosen. Amoxicillin (AML 10 µg), gentamycin (CN 10 µg), cefixime (CFM 5 µg), cefotaxime (CTX 30 µg), doxycycline (Do 30 µg), cephalothin (KF 30 µg), and sulfisoxazole (300 µg). An inoculated 24 h broth with visible turbidity that is equal to or greater than that of the 0.5 McFarland Standard was used. An inoculum was spread evenly over the entire surface of dry Iso-Sensitest agar plate (ISA; Oxoid, Basingstoke, UK) by swabbing in three directions. The inoculum was semi-confluent with no gaps between the swab streaks. Discs were applied to the surface of the agar within 15 min of inoculation. The plate was inverted and incubated at 37°C for 24-48 h. The diameters of zones of inhibition to the nearest millimeters were measured with a graduated ruler and were translated to sensitive, intermediate, and resistant categories according to the guidelines of Andrews [34].

### Measurement of multiple antibiotic resistance (MAR) index

The MAR index for STEC isolates was calculated according to the formula of Krumperman [35]: MAR = a/b, where a is a number of antibiotics to which the isolate was resistant, b is a number of antibiotics to which the isolate was subjected. Intermediate and resistant categories were both considered resistant when calculating MAR as suggested by Luczkiewicz *et al*. [36].

### DNA extraction

The suspect STEC isolates were cultured on nutrient agar. A single colony was used for PCR. The DNA was isolated from a colony sweep by suspending it in 500 µl of sterile Milli-Q water. The suspension was boiled for 10 min at 95°C and centrifuged at 10,000 × *g* for 10 min. The supernatant was then used as the DNA template [37].

### Multiplex PCR assay

The multiplex PCR assays were standardized for the detection of *stx*_1_, *stx*_2_ [38], and *eae* genes [39]. The DNA templates were subjected to multiplex PCR with the following primers: *stx*_1_ F: 5’CTG GAT TTA ATG TCG CAT AGT G3’, *stx*_1_ R: 5’AGA ACG CCC ACT GAG ATC ATC3’, *stx_2_* F: 5’ GGC ACT GTC TGA AAC TGC TCC 3’ and *stx*_2_ R: 5’ TCG CCA GTT ATC TGA CAT TCT G 3’, *eae* F: 5’ GAC CCG GCA CAA GCA TAA GC 3’, and *eae* R: 5’ CCA CCT GCA GCA ACA AGA GG 3’ yielding 150, 255 and 384 (bp) products, respectively. The primers were ordered from Bio basic Inc., Canada. The specificity of each primer was confirmed by monoplex PCR. The total reaction volume was 25 µl containing 5 µl of the extracted DNA from STEC isolates, 12.5 µl of 2× PCR master mix (GeneDirex, USA and Taiwan), 0.5 µl of each primer (20 pmol), and 4.5 µl of sterile Milli-Q water. An Applied Biosystems GeneAmp^®^ PCR System was used for the PCR thermal cycling conditions with an initial denaturation step at 95°C for 3 min, 35 cycles (denaturation at 95°C for 1 min, annealing at 57°C for 1 min, extension at 72°C for 1 min) and a final extension step at 72°C for 7 min. The amplified products were then run along a 0.1-0.5 µg/ml ethidium bromide-stained agarose gel 1.5% with a 100 bp DNA ladder (GeneDirex, USA and Taiwan) in 1× TBE buffer for 30 min at 100 V and then recorded using the SynGene Gel Documentation System.

### RAPD PCR assay

Preliminary trials were done using 10 random decamer oligonucleotide primers (OPA1-OPA 10, Eurofins Genomics, Brussels - Belgium) and different PCR conditions. The most yielding primers for discrimination of STEC were OPA-2 (5’TGCCGAGCTG3’), OPA-9 (5’GGGTAACGCC3’) followed by OPA-10 (5’GTGATCGCAG3’) (data were not shown). The most optimum PCR condition was that described by Hopkins and Hilton [40] with modifications. The PCR was carried out in a 25-µl volume containing 12.5 µl PCR master mix (GeneDirex, USA, and Taiwan), 1 µl of primer (OPA-2, OPA-9, or OPA-10) (30 pmol), 3 µl of DNA and 8.5 µl of sterile Milli-Q water. The reactions were run using Techne thermal cycler (Techne, Cambridge, UK). The reactions consisted of one cycle of 4.5 min at 94°C followed by five low stringency cycles of 30 s at 94°C, 1 min at 22°C, 2 min at 72°C and 35 high stringency cycles of 30 s at 94°C, 30 s at 28°C, and 3 min at 72°C. A final extension at 72°C for 5 min and the reactions were hold at 4°C until analysis. The amplified products (8 µl) were separated by electrophoresis in 1.5% agarose stained with ethidium bromide (0.1-0.5 µg/ml) with two DNA ladders (100-1500 bp and 100-3000 bp) and recorded using the SynGene Gel Documentation System.

### Analysis of RAPD data

RAPD data were analyzed using computer software (SynGene GeneTools - File version: 4.03.05.0). A scoring Excel sheet was made. Each isolate was scored for the presence (1) or absence (0) of each band on agarose gel. Different banding patterns were recorded. A difference of >2 bands were considered different strains, while isolates with ≤2 bands difference were regarded as the same strain [30].

### Statistical analysis

The percentages of colonization were compared using Chi-square test, using SPSS version (20). The p value was set at p≤0.05. The molecular relatedness and genotypic clustering of isolates were analyzed by converting the data to binary code, creating triangular similarity matrix and dendrograms using primer (5) software.

## Results

The bacteriological analysis indicated that the percentages of *E. coli* and STEC colonization were highest in pigeons (90.6 and 46.9), followed by cattle egrets (44.4 and 16.7), crows (41.7 and 9.5), moorhens (39.4 and 6.1), and house teals (20 and 20), respectively (Table-1). Chi-square values for *E. coli* and STEC colonization were χ2=28.723 and χ2=26.496, at p<0.0001, respectively. Both were considered significant.

**Table-1:** Distribution of STEC serotypes among wild bird species.

Species	Ex. No.	n (%)		STEC serotypes n (%)													
	
		Positive *E. coli*	Positive STEC	O:20	O:25	O:26	O:27	O:63	O:78	O:111	O:114	O:125	O:128	O:142	O:153	O:158	Rough
Cattle egrets	18	8 (44.4)	3 (16.7)	-	-	-	1 (33.3)	-	-	1 (33.3)	-	-	-	-	-	-	1 (33.3)
Pigeons	32	29 (90.6)	15 (46.9)	1 (6.7)	-	-	4 (26.7)	1 (6.7)	1 (6.7)	-	1 (6.7)	-	1 (6.7)	-	-	4 (26.7)	2 (13.3)
Crows	84	35 (41.7)	8 (9.5)	-	1 (12.5)	-	-	-	-	-	2 (25)	2 (25)	-	1 (12.5)	1 (12.5)	-	1 (12.5)
Moorhens	33	13 (39.4)	2 (6.1)	-	-	-	1 (50)	-	-	-	-	-	-	-	-	-	1 (50)
House teals	10	2 (20)	2 (20)	-	-	1 (50)	1 (50)	-	-	-	-	-	-	-	-	-	-
Total no.	177	87	30	1	1	1	7	1	1	1	3	2	1	1	1	4	5

For *E. coli*, χ^2^=28.723, p<0.0001. For STEC, χ^2^=26.496, p<0.0001. *E. coli=Escherichia coli*, STEC=Shiga toxin producing *Escherichia coli*

The data that were shown in Table-1 revealed that 30 suspect STEC isolates were recovered from (30) positive birds’ samples. By serotyping, (25) isolates were identified as follows: From pigeons, serotypes (O20, [1]; O27, [4]; O63, [1]; O78, [1]; O114 [1]; O128, [1]; and O158, [4]) were detected. The isolates from crows were identified as O25, (1); O114, (2); O125 (2); O142, (1); and O153, (1). Two serotypes were recovered from cattle egrets (O27 and O111), one from moorhens (O27), and two from house teals (O26 and O27). In addition to, five rough strains. However, none of the STEC isolates belonged to the O157 serogroup.

PCR screening of the virulence genes of STEC revealed that *stx*_1_ gene only was detected in 16 birds as follows: (28.1%, 9/32) from pigeons, (7.1%, 6/84) from crows, in addition to (5.6%, 1/18) from cattle egrets. There were four birds that possessed both *stx*_1_ and *stx*_2_ genes in the following manner: (5.6%, 1/18) from cattle egrets, (6.1%, 2/33) from moorhens, and (10%, 1/10) from house teals. Only one pigeon (1/32, 3.1%) possessed the three alleles (Data were retrieved from Table-2).

**Table-2:** Epidemiologic data, phenotypic and genotypic traits of STEC serotypes.

Serial number	Code	Source	Serotype	Antibiotic type	Phenotypic pattern							a	MAR index	Source	Serotype	Virulence factors			RAPD patterns	
		
					A	G	C	CT	D	K	S					*stx_1_*	*stx_2_*	*eae*	Primer 2 A	Primer 9 B
17 P	9E	Pigeon	O27	Type 2	R	R	S	R	R	R	R	6	0.857	Pigeon	O27	-	-	-	A9	B9
13 P	8E	Pigeon	O27	Type 2										Pigeon	O27	+	-	-	A8	B8
3 P	24E	Pigeon	O114	Type 3	R	S	S	R	R	R	R	5	0.714	Pigeon	O114	-	-	-	A24	-
16 p	10E	Pigeon	O78	Type 3										Pigeon	O78	+	-	-	A10	B10
21 P	7E	Pigeon	O27	Type 3										Pigeon	O27	+	-		A7	B7
11 P	4E	Pigeon	Rough	Type 4	I	R	S	R	R	R	R	6	0.857	Pigeon	Rough	+	+	+	A4	B4
7 P	3E	Pigeon	O128	Type 4										Pigeon	O128	+	-	-	A3	B3
2 P	5E	Pigeon	O20	Type 6	S	R	S	R	R	R	R	5	0.714	Pigeon	O20	-	-	-	A5	B5
18 P	6E	Pigeon	O27	Type 6										Pigeon	O27	-	-	-	A6	B6
31 P	16E	Pigeon	O63	Type 8	S	S	S	R	R	R	R	4	0.571	Pigeon	O63	+	-	-	A16	B16
5 P	12E	Pigeon	O158	Type 8										Pigeon	O158	+	-	-	A12	B12
10 p	11E	Pigeon	O158	Type 8										Pigeon	O158	+	-	-	A11	B11
29 P	14E	Pigeon	O158	Type 8										Pigeon	O158	-	-	-	A14	B14
30 P	15E	Pigeon	O158	Type 8										Pigeon	O158	+	-	-	A15	B15
28 P	13E	Pigeon	Rough	Type 8										Pigeon	Rough	+	-	-	A13	B13
5 E_g_	2E	Cattle egret	O27	Type 2										Cattle egret	O27	+	+	-	A2	B2
7 E_g_	1E	Cattle egret	O111	Type 6										Cattle egret	O111	-	-	-	A1	B1
11 E_g_	29E	Cattle egret	Rough	Type 7	S	S	R	R	R	R	R	5	0.714	Cattle egret	Rough	+	-	-	A29	B26
76 C	25E	Crow	O125	Type 2										Crow	O125	+	-	-	A25	B22
42 C	17E	Crow	O114	Type 3										Crow	O114	+	-	-	A17	B17
80 C	27E	Crow	O 153	Type 3										Crow	O 153	-	-	-	A27	B24
84 C	28E	Crow	O25	Type 5	I	S	S	R	R	R	R	5	0.714	Crow	O25	+	-	-	A28	B25
81 C	30E	Crow	O125	Type 6										81 C	Crow	O125	-	-	-	A30	B27
71 C	23E	Crow	O114	Type 8										71 C	Crow	O114	+	-	-	A23	B21
32 C	22E	Crow	O142	Type 8										32 C	Crow	O142	+	-	-	A22	-
48 C	18E	Crow	Rough	Type 8										Crow	Rough	+	-	-	A18	B18
8 M	19E	Moorhen	O27	Type 2										8 M	Moorhen	O27	+	+	-	A19	B19
9 M	26E	Moorhen	Rough	Type 6										Moorhen	Rough	+	+	-	A26	B23
5 T	20E	House teal	O27	Type 1	R	R	R	R	R	R	R	7	1	House teal	O27	-	-	-	A20	B20
6 T	21E	House teal	O26	Type 3										House teal	O26	+	+	-	A21	-

A=Amoxicillin. G=Gentamycin, C=Cefixime, CT=Cefotaxime, D=Doxycycline, K=Cephalothin, S=Sulfisoxazole. Total number of antibiotics to which the isolate is resistant (a). Total number of antibiotics to which the isolate is subjected (b)=7. MAR index=a/b

Positive growth on STEC CHROMagar that was confirmed by *stx* gene-detecting PCR indicated that the specificity of STEC CHROMagar medium for detecting STEC was (70%), as nine non-*stx*-producing isolates out of (30) STEC isolates grew as mauve colonies.

The antibiotic susceptibility patterns showed that cefixime was the most effective against STEC serotypes with 93.3% sensitivity and 6.7% resistance, while the sensitivity and resistance percentages of gentamycin were 56.7 and 43.3 and for amoxicillin were 50 and 40, respectively. All the recovered serotypes were resistant to cefotaxime, doxycycline, cephalothin, and sulfisoxazole (Table-3). The recovered STEC serotypes were organized into eight phenotypic groups according to their antibiogram susceptibility patterns. 30% of STEC isolates (n=9) belonged to phenotype (8); they were resistant to four antibiotics and had MAR index of 0.571. Types 3, 5, 6, and 7 comprised (43.3%, n=13) of STEC isolates; they were resistant to five antibiotics and had MAR index of 0.714. Types 2 and 4 (23.3% of STEC, n=7) were resistant to six antibiotics, having MAR index of 0.857. Type 1 was resistant to all antibiotics tested (Table-2).

**Table-3:** Antibiogram susceptibility pattern of different STEC serotypes.

Antibiotic tested	STEC serotype														

	O:20	O:25	O:26	O:27	O:63	O:78	O:111	O:114	O:125	O:128	O:142	O:153	O:158	Rough	Total
														
	S (%) I (%) R (%)	S (%) I (%) R (%)	S (%) I (%) R (%)	S (%) I (%) R (%)	S (%) I (%) R (%)	S (%) I (%) R (%)	S (%) I (%) R (%)	S (%) I (%) R (%)	S (%) I (%) R (%)	S (%) I (%) R (%)	S (%) I (%) R (%)	S (%) I (%) R (%)	S (%) I (%) R (%)	S (%) I (%) R (%)	S (%) I (%) R (%)
Amoxicillin	1 (6.7)	-	-	1 (6.7)	1 (6.7)	-	1 (6.7)	1 (6.7)	1 (6.7)	-	1 (6.7)	-	4 (26.7)	4 (26.7)	15 (50)
	-	1 (33.3)	-	-	-	-	-	-	-	1 (33.3)	-	-	-	1 (33.3)	3 (10)
	-	-	1 (8.3)	6 (50)	-	1 (8.3)	-	2 (16.7)	1 (8.3)	-	-	1 (8.3)	-	-	12 (40)
Gentamycin	-	1 (5.9)	1 (5.9)	1 (5.9)	1 (5.9)	1 (5.9)	-	3 (17.6)	-	-	1 (5.9)	1 (5.9)	4 (23.5)	3 (17.6)	17 (56.7)
	-	-	-	-	-	-	-	-	-	-	-	-	-	-	-
	1 (7.7)	-	-	6 (46.2)	-	-	1 (7.7)	-	2 (15.4)	1 (7.7)	-	-	-	2 (15.4)	13 (43.3)
Cefixime	1 (3.6)	1 (3.6)	1 (3.6)	6 (21.4)	1 (3.6)	1 (3.6)	1 (3.6)	3 (10.7)	2 (7.1)	1 (3.6)	1 (3.6)	1 (3.6)	4 (14.3)	4 (14.3)	28 (93.3)
	-	-	-	-	-	-	-	-	-	-	-	-	-	-	-
	-	-	-	1 (50)	-	-	-	-	-	-	-	-	-	1 (50)	2 (6.7)
Cefotaxime	-	-	-	-	-	-	-	-	-	-	-	-	-	-	-
	-	-	-	-	-	-	-	-	-	-	-	-	-	-	-
	1 (3.3)	1 (3.3)	1 (3.3)	7 (23.3)	1 (3.3)	1 (3.3)	1 (3.3)	3 (10)	2 (6.7)	1 (3.3)	1 (3.3)	1 (3.3)	4 (13.3)	5 (16.7)	30 (100)
Doxycycline	-	-	-	-	-	-	-	-	-	-	-	-	-	-	-
	-	-	-	-	-	-	-	-	-	-	-	-	-	-	-
	1 (3.3)	1 (3.3)	1 (3.3)	7 (23.3)	1 (3.3)	1 (3.3)	1 (3.3)	3 (10)	2 (6.7)	1 (3.3)	1 (3.3)	1 (3.3)	4 (13.3)	5 (16.7)	30 (100)
Cephalothin	-	-	-	-	-	-	-	-	-	-	-	-	-	-	-
	-	-	-	-	-	-	-	-	-	-	-	-	-	-	-
	1 (3.3)	1 (3.3)	1 (3.3)	7 (23.3)	1 (3.3)	1 (3.3)	1 (3.3)	3 (10)	2 (6.7)	1 (3.3)	1 (3.3)	1 (3.3)	4 (13.3)	5 (16.7)	30 (100)
Sulfisoxazole	-	-	-	-	-	-	-	-	-	-	-	-	-	-	-
	-	-	-	-	-	-	-	-	-	-	-	-	-	-	-
	1 (3.3)	1 (3.3)	1 (3.3)	7 (23.3)	1 (3.3)	1 (3.3)	1 (3.3)	3 (10)	2 (6.7)	1 (3.3)	1 (3.3)	1 (3.3)	4 (13.3)	5 (16.7)	30 (100)
Total examined	1	1	1	7	1	1	1	3	2	1	1	1	4	5	30

S=Sensitive, I=Intermediate, R=Resistant

**Figure-1:**
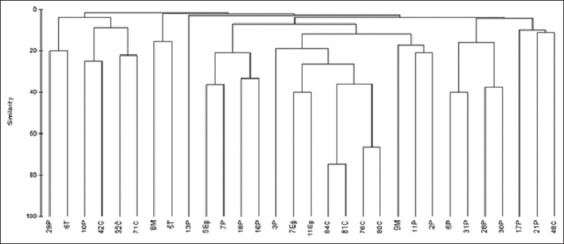
Dendrogram based on random amplified polymorphic DNA profile of Shiga toxin producing *Escherichia coli* using primer OPA-2.

The computer aided RAPD PCR analysis using (OPA-2) primer yielded 122 different bands. The molecular masses of the fragments ranged between 133 and 1777 bp. Primer (OPA-2) could differentiate all the STEC strains from one another yielding 30 different RAPD profiles. At 80% similarity, the 30 STEC isolates lied into 30 different clusters (Figure-1). Using (OPA-9), a total of 27 RAPD profiles were observed. 94 bands ranged between 156 and 1997 bp were distinguished among the 27 STEC isolates. At 80% similarity, the STEC isolates yielded 27 different clusters (Data were not shown). Primer (OPA-9) was unable to amplify serotypes O 26 from teals, O 142 from crows and O 114 from pigeons. Primer (OPA-10) could not amplify most of the isolates (Data were not shown).

## Discussion

Zoonoses with a wildlife reservoir represent a global public health problem [41]. The migratory nature of birds, their ability to cover vast distances within a relatively short period of time, their residence near livestock areas, farms, waste disposal sites, and human habitats made them important vectors of some zoonoses [7,23,42]. Human infections with STEC are increasingly recognized as causes of HC and HUS [10]. The most renowned example is *E. coli* O157:H7, however the prevalence of non-O157 STEC can be the same or even exceeds that of O157: H7 [6]. Among patients with non-O157 STEC infections, the “Big Six” strains; O26, O45, O103, O111, O121, and O145 are the most common cause of hospitalizations. These types can cause the most severe sequelae as kidney failure (HUS) and death [43]. The incidence of DEC can vary from one region to another due to varying principles and methods for detection [2]. For example, the annual detection rate of non-O157 STEC infection in the USA exceeds that caused by O157 STEC [5]. In developing countries, the detection and identification of non-O157 STEC infections are complex and underestimated. First, most efforts are directed to detect O157. Yet, non-O157 STEC is not recognized on the media that are used to isolate this organism. Second, clinical laboratories must detect Shiga toxins in stool samples and then, the positive samples must be sent to the public health laboratories for serotyping [44]. The financial cost is another keystone factor that may hinder such diagnosis.

Earlier studies confirmed the importance of wild birds in the maintenance of *E. coli*. In Germany, *E. coli* was isolated from birds of prey, waterfowls and passerines [24]. In Denmark, the frequency of VTEC-positive wild birds was 1.6%, as judged by the PCR screening, showing that wild birds may become infected from farm animals or vice versa [13]. Foster *et al*. [14] reported that one (0.43%) out of 231 composite feces collected from a bird table in southwest Scotland was positive for *E. coli* O157. They attributed the low isolation rate to either the storage process, the sensitivity of the test method, or other factors. Kobayashi *et al*. [17] recorded that the prevalences of *stx-* and *eae*-positive strains of *E. coli* among wild birds in Japan were 5% (23/447) and 25% (113/447), respectively. In Egypt, *E. coli* was isolated from white ibis at the rate of 43.6% [45]. In Germany, *E. coli* was the second most common isolated pathogen, being present in 18% (46/251) of diseased wild birds [19]. Koochakzadeh *et al*. [20] found that 0.45% of wild and pet birds in Iran carry EPEC strains. Gioia-Di Chiacchio *et al*. [7] found that *E. coli* isolated from psittacine birds in Brazil was *eae*^+^ and *stx_2_*^+^.

*Stx*-PCR confirmed results revealed that the specificity of STEC CHROMagar medium for detecting STEC was (70%). However, STEC isolates might contain other *stx* variants, and this needs further investigation.

The main virulence factor of STEC group is the production of *stx*_1_ or *stx*_2_ proteins. It is worth mentioning that Byrne *et al*. [46] reported that HUS is significantly associated with STEC strains possessing *eae* and/or *stx_2_*. In this study, most of the isolates possessed *stx*_1_ gene. The significance of *stx*_1_ was underscored by Hedican *et al*. [6] who indicated that non-O157 isolates that had only *stx*_1_ can cause severe bloody diarrhea or HUS. Furthermore, Käppeli *et al*. [4] depicted the *stx* gene distribution among 97 non-O157 STEC strains isolated from HUS cases in Switzerland. They showed that 45 (46.4%) strains had only *stx*_2_, 36 (37.1%) had only *stx*_1_, and 16 (16.5%) had both genes.

In contrast, other researchers could not detect *stx*_1_ or *stx*_2_ in wild birds. Kobayashi *et al*. [16] found no *stx*^+^ samples among fecal samples from gulls, pigeons, and chickens that were examined in Finland. Koochakzadeh *et al*. [20] found that 1.8% of wild and pet birds carry *stx_2f_*^+^ STEC. Schmidt *et al*. [47] in their study in Italy reported the isolation of STEC strains from the feces of feral pigeons which contained a new *stx*_2_ variant gene designated *stx*_2f_. Wani *et al*. [48] showed that none of the isolated *E. coli* strains (O9, O18, O25, O60, O77, O147, O157, O168, O169, rough [R], untypable [UT]) from free flying pigeons in India were *stx*_1_^+^ or *stx*_2_^+^.

The current research develops the claim that pigeons can be a source of STEC for humans. Among the examined wild bird species, they had the highest STEC colonization rate (46.9%) of which 10/32, 31.3% carried virulence genes. They were also colonized with a diverse number of STEC serotypes. It is noteworthy to mention that pigeons are commonly found in human residence areas and parks, moreover, they are commonly raised and/or consumed in Egypt; an issue that may allow such transmission. Along similar lines, Pedersen and Clark [23] reported that pigeons and sparrows are important in recirculation of STEC. In Italy, *stx* genes were detected in 10.8% of the stool enrichment cultures collected from feral pigeons [42]. In another study in Italy [9], four *E. coli* O157:H7 strains from pigeons were isolated; all strains carried *eae* and *stx_2_* genes, whereas only one strain carried the *stx*_1_ gene. In Germany, 67% of the examined pigeon feces harbored *stx* genes [49]. Dutta *et al*. [12] found that out of 150 pigeons subjected to microbiological investigation in India, 91 (60.67%) samples were found positive for *E. coli*. The most frequently occurring serotypes were O157, followed by O68, O121, O9, O75, O131, O2, O13, and O22.

Concerning crows, cattle egrets and water fowls, they can contaminate water and pasture with their fecal droppings; moreover, they have been implicated as a source of DEC infection [15,44,45]. The noteworthy point is that people living in Egyptian coastal cities are accustomed to eating different spp. of wild water fowls. They purchase them either eviscerated or even process them at home. Thus, the increased prevalence of STEC colonization in moorhens (6.1%) and house teals (20%) may represent a hazard to human contacts. The following section will highlight the importance of wild birds as indicators, long-distance vectors, reservoirs, and potential spreaders of MDR *E. coli* and this was previously confirmed by various researchers [18,24-28,50]. In this study, STEC had MAR index that ranged from 0.571 to 1. MAR indexing has been used as an indicator to identify high-risk contamination that may pose a hazard to humans. MAR index values which are higher than 0.2 were considered to have originated from high-risk sources where antibiotics are often used [35]. Evidence for antibiotic-resistant *E. coli* was borne out by many researchers. Guenther *et al*. [24] showed that nine of the 187 *E. coli* isolates (4.8%) exhibited multiresistant phenotypes including resistances against beta-lactams, aminoglycosides, fluoroquinolones, tetracyclines, and sulfonamides. Similarly, Käppeli *et al*. [4] investigated the antimicrobial pattern of non-O157 STEC strains isolated from HUS cases in Switzerland; all the recovered strains were susceptible to five antimicrobial drugs (ceftazidime, ciprofloxacin, cefotaxime, cefepime, and cefoxitin). In Spain, 41% out of 581 non-O157 STEC strains showed resistance to at least one of the 26 antimicrobial agents tested and sulfisoxazole (36%) had the most common antimicrobial resistance [51]. They found also an association between a higher level of multiple resistances to antibiotics and the presence of the virulence genes *eae* and *stx*_1_ among non-O157 strains. Hasan *et al.’s* [18] results provided confirmatory evidence that wild ducks and domestic poultry harbored the same ESBL-producing *E. coli* isolates. They attributed this commonality to be caused by a common use of natural water resources. They concluded also that *E. coli* that produces CTX-M-15 is endemic to birds in Bangladesh. Similarly, Dutta *et al*. [12] concluded that antimicrobial-resistant pathogenic *E. coli* is present in pigeons. In Spain, cefotaxime-resistant *E. coli* isolates were identified in 16 of the 100 tested wild birds’ species samples [28]. In summary, the present study reveals that a high percentage of wild birds carry antibiotic-resistant STEC and this may pose a threat to human and animal health.

The importance of typing methods for zoonotic disease prevention and control is increasingly esteemed. One of the useful means that is used for discrimination of bacterial strains within the same spp. is RAPD Analysis. The usefulness of RAPD PCR for tracing the clonal relations of *E. coli* serovars has been underscored by several researchers [30,40,52,53]. The results of our study demonstrated that RAPD PCR analysis of the *E. coli* strains in conjunction with serotyping may fulfill these criteria. The use of computerized analysis aided in the differentiation of 30 and 27 different DNA fingerprinting profiles for primers OPA-2 and OPA-9, respectively. Most of these profile bands were impossible to be correctly sized using naked eye analysis. Correspondingly, in India, primers OPAC 04, OPAC 07, OPAC 09, OPAC 11, and OPAC 12 yielded entirely different banding pattern for each *E. coli* serotype and were able to differentiate all the serotypes from one another [52]. The view that at least two independent primers should be used to maximize the discriminatory capacity of RAPD PCR is in line with Idil and Bilkay [54]. There are some previous reports on RAPD genotyping of *E. coli* strains using one or two primers and agarose electrophoresis, but none of these studies have used computer-aided analysis of bands [55,56]. Our results showed that non-O157 STEC are heterogeneous; they were grouped into 30 different clusters at 80% similarity. The finding that certain isolates could not be amplified using OPA-9 and OPA-10 primers might be attributed to the absence of sequence in the bacterial DNA which is complementary to the sequence of the primer and the fact that some primers may amplify only a small sequence of the genome, while other primers may amplify different sites of the genome and so differentiate between strains [53,56].

Overall, the investigated birds which were sampled from live bird markets, parks and areas near human residence areas carry STEC that harbor virulence genes and are resistant to multiple antibiotics. We also found that RAPD PCR when complemented with serotyping become useful means for discrimination of STEC strains.

## Conclusions

Wild birds must be monitored for MDR zoonotic pathogens including STEC. Therefore, the development of local centers that are globally connected for the early detection, prevention, and control of such infections must be prioritized.

## Authors’ Contributions

This research was designed and funded by HMF and RA. HMF was responsible for collection of samples, performance of the experiments, writing and revising of the manuscript. RA collected samples and shared in isolation and identification of *E. coli*. DMA performed PCR for detection of *stx*_1_, *stx*_2_, and *eae* genes. The manuscript has been seen and approved by all authors.
